# Diversity of antibiotic resistance gene variants at subsequent stages of the wastewater treatment process revealed by a metagenomic analysis of PCR amplicons

**DOI:** 10.3389/fgene.2023.1334646

**Published:** 2024-01-11

**Authors:** Adrian Gorecki, Piotr Ostapczuk, Lukasz Dziewit

**Affiliations:** ^1^ Department of Biochemistry and Microbiology, Institute of Biology, Warsaw University of Life Sciences (SGGW), Warsaw, Poland; ^2^ Department of Environmental Microbiology and Biotechnology, Institute of Microbiology, Faculty of Biology, University of Warsaw, Warsaw, Poland

**Keywords:** antibiotic resistance gene, antibiotic resistance gene amplicon, antibiotic resistant bacteria, wastewater treatment plant, PCR diversification power

## Abstract

Wastewater treatment plants have been recognised as point sources of various antibiotic-resistant bacteria (ARB) and antibiotic resistance genes (ARG) which are considered recently emerging biological contaminants. So far, culture-based and molecular-based methods have been successfully applied to monitor antimicrobial resistance (AMR) in WWTPs. However, the methods applied do not permit the comprehensive identification of the true diversity of ARGs. In this study we applied next-generation sequencing for a metagenomic analysis of PCR amplicons of ARGs from the subsequent stages of the analysed WWTP. The presence of 14 genes conferring resistance to different antibiotic families was screened by PCR. In the next step, three genes were selected for detailed analysis of changes of the profile of ARG variants along the process. A relative abundance of 79 variants was analysed. The highest diversity was revealed in the *ermF* gene, with 52 variants. The relative abundance of some variants changed along the purification process, and some ARG variants might be present in novel hosts for which they were currently unassigned. Additionally, we identified a pool of novel ARG variants present in the studied WWTP. Overall, the results obtained indicated that the applied method is sufficient for analysing ARG variant diversity.

## 1 Introduction

Antimicrobial resistance (AMR) is considered to be one of the most serious threats to public health and has become a major concern for national governments and international organisations (O’Neil, 2014). This is mainly due to constantly increasing market demands coupled with poorly-controlled utilisation and usage of antibiotics (Alanis, 2005). Since their initial introduction 60 years ago, millions of metric tons of antibiotics have been used in a variety of applications (Davies and Davies, 2010). Additionally, there is plenty of evidence of the overuse and inappropriate prescription of antibiotics in the fields of medicine and veterinary medicine. Studies have shown that between 30% and 60% of antibiotics prescribed are incorrect in choice, indication or duration of treatment (Luyt et al., 2014). Such circumstances provoke the selection of opportunistic multi-resistant bacteria and novel antibiotic resistance genes (ARGs). Wastewater treatment plants (WWTPs) are considered a point source of significantly accumulated diverse contaminants, including: i) mobile genetic elements (MGEs) as potential vectors of genes (e.g., ARGs) impacting bacterial fitness under various environmental conditions, ii) heavy metals and iii) emerging pollutants (e.g., antibiotics, microplastics, nanomaterials, pharmaceuticals and personal care products and quorum sensing inhibitors), that can impact the proliferation of ARGs and antibiotic resistant bacteria (ARB). WWTPs are also one of the most important interfaces between the human population and the environment (Gao et al., 2022).

There are multiple techniques available to monitor ARB and ARGs in WWTPs (Wengenroth et al., 2021). These include culture-based and molecular-based (culture-independent) approaches. Culture-dependent methods are usually used to identify phenotypes of specific bacterial taxa, including coliform bacteria and enterococci. The most commonly used cultured-based methods are dilution tests, disk diffusion tests, E-tests and automated systems, such as VITEK2. However, since less than 10% of known bacterial species can be cultivated (Dziurzynski et al., 2023), culture-based methods are strongly biased and leave considerable amounts of information concerning the environmental dimension of AMR unexplored. In the second approach, molecular-based techniques allow detailed insight into a non-cultivable fraction of microbiota, with a special focus on diversity and prevalence of particular genes, e.g., ARGs. Commonly used molecular monitoring methods include: PCR, multiplex PCR, quantitative PCR, shotgun sequencing or microarrays (Miłobedzka et al., 2021). In those approaches there are also some limitations of which the uncertainty about the functionality and completeness of identified genes and bias towards the most abundant variants of searched ARG are the most important issues.

In this study, next-generation sequencing (NGS) was applied for the generation of ARG-amplicon metagenomic data. They were produced for three selected genes (i.e., *ermF*, *sul2* and *tetX*) conferring resistance to macrolides, sulfonamides and tetracyclines, respectively. These genes were detected at all stages of the purification process in the studied WWTP. Subsequent bioinformatic analysis showed the internal heterogeneity of generated PCR amplicons and revealed a high diversity of ARG variants. To the best of our knowledge, this is the first study showing the diversity of ARG variants at subsequent stages of a WWTP.

## 2 Materials and methods

### 2.1 Characterisation of samples and sampling location

All samples were collected from the municipal and industrial WWTP located in Oswiecim (Poland) in October 2016. The criteria for the selection of this WWTP were as follows: i) relatively large capacity (of about 195 m^3^ of wastewater per day); ii) mixed wastes (including communal and industrial wastes); iii) dividing of the treatment process into three main stages, i.e., primary treatment (PS), secondary treatment (AS) and anaerobic digestion (ADS). The sludge samples (5 L from each above-mentioned stage) were collected into sterile glass bottles and transferred directly to the laboratory. In the laboratory, the samples were distributed into sterile 50 mL Falcon™ tubes and centrifuged for 10 min at 5,900 g. After the supernatant was removed, the samples were procced for DNA extraction.

### 2.2 DNA extraction

Total DNA was extracted from 500 mg of pellet obtained from raw sludge using a Fast DNA Spin Kit for Feces (MP Biomedicals, Illkirch, France) in accordance with the manufacturer’s instructions. The DNA concentration was determined using the Qubit™ 2.0 Fluorometer (Invitrogen, Carlsbad, CA, United States).

### 2.3 Screening of ARGs in samples from the WWTP

The occurrence of ARGs in the analysed samples was investigated using PCR assays. The PCRs were performed in triplicates with 10 pmol of each primer (Table 1), DreamTaq PCR Master Mix (Thermo Fisher Scientific, Waltham, MA, United States) and 10 ng of the DNA template. The PCR conditions changed as per the recommended annealing temperature for each primer pair (Table 1). The amplification of all genes began with denaturation at 95°C for 3 min. Denaturation was followed by 30 cycles of 30 s at 98°C, 30 s at the annealing temperature (Table 1), and 30 s of extension at 72°C. A final extension of 72°C for 10 min was used for all samples. The PCR products were resolved by electrophoresis in 1.50% agarose gel. PCR products of the correct size were excised from the gel after electrophoresis, purified using an Agarose-Out DNA purification Kit (EURx, Gdansk, Poland) and then ligated to vector pNZY28-A (Nzytech, Lisbon, Portugal) and introduced into *Escherichia coli* DH5α (Hanahan, 1983) via chemical transformation (Kushner, 1978). The DNA of recombinant plasmids carrying the cloned PCR products was purified using a Plasmid Miniprep DNA Purification Kit (EURx, Gdansk, Poland) and the inserts of three clones were sequenced using universal primers (M13/pUC) with an ABI-PRISM 377 capillary DNA analyser (Applied Biosystem, Foster City, CA, United States) at the Genomed company (Poland).

**TABLE 1:** Primer pairs used in this study.

Target gene	Forward primer (5′ to 3′)	Reverse primer (5′ to 3′)	PCR product size (bp)	Annealing temperature (°C)	References
***aadB***	ANT2-FW: ATCTGCCGCTCTGGAT	ANT2-RV: CGAGCCTGTAGGACT	405	44.70	This study
***ermB***	ERMB-FW: GGT​TGC​TCT​TGC​ACA​CTC​AAG	ERMB-RV: CAG​TTG​ACG​ATA​TTC​TCG​ATT​G	191	51.10	Koike et al. (2010)
***ermF***	ERMF-FW: TCT​GGG​AGG​TTC​CAT​TGT​CC	ERMF-RV: TTC​AGG​GAC​AAC​TTC​CAG​C	424	51.10	Koike et al. (2010)
***qrnB***	QNRB-FW: GAT​CGT​GAA​AGC​CAG​AAA​GG	QNRB-RV: ACG​ATG​CCT​GGT​AGT​TGT​CC	469	51.80	Robicsek et al. (2006)
***sul1***	SUL1-FW: CGG​CGT​GGG​CTA​CCT​GAA​CG	SUL1-RV: GCC​GAT​CGC​GTG​AAG​TTC​CG	432	57.90	Arabi et al. (2015)
***sul2***	SUL2-FW: GCG​CTC​AAG​GCA​GAT​GGC​ATT	SUL2-RV: GCG​TTT​GAT​ACC​GGC​ACC​CGT	293	56.30	Arabi et al. (2015)
***tetA***	TETA-FW: GTA​ATT​CTG​AGC​ACT​GTC​GC	TETA-RV: CTG​CCT​GGA​CAA​CAT​TGC​TT	957	51.80	Guardabassi et al. (2000)
***tetB***	TETB-FW: ACA​CTC​AGT​ATT​CCA​AGC​CTT​TG	TETB-RV: GAT​AGA​CAT​CAC​TCC​CTG​TAA​TGC	205	53.50	Peak et al. (2007)
***tetC***	TETC-FW: CTT​GAG​AGC​CTT​CAA​CCC​AG	TETC-RV: ATG​GTC​GTC​ATC​TAC​CTG​CC	418	53.80	Ng et al. (2001)
***tetG***	TETG-FW: TTA​TCG​CCG​CCG​CCC​TTC​T	TETG-RV: TCA​TCC​AGC​CGT​AAC​AGA​AC	133	51.80	Song et al. (2021)
***tetM***	TETM-FW: GTG​GAC​AAA​GGT​ACA​ACG​AG	TETM-RV: CGG​TAA​AGT​TCG​TCA​CAC​AC	406	51.80	Ng et al. (2001)
***tetO***	TETO-FW: GAT​GGC​ATA​CAG​GCA​CAG​ACC	TETM-RV: GCC​CAA​CCT​TTT​GCT​TCA​CTA	172	52.40	Luo et al. (2010)
***tetT***	TETT-FW: AAC​GGA​TTC​GAT​GGA​ACT​TG	TETT-RV: GGA​CTT​GAA​TTC​CTT​CTT​TTG​C	199	49.70	Szczepanowski et al. (2009)
***tetX***	TETX-FW: CAA​TAA​TTG​GTG​GTG​GAC​CC	TETX-RV: TTC​TTA​CCT​TGG​ACA​TCC​CG	468	51.80	Ng et al. (2001)

### 2.4 Taxonomic classification of 16S rDNA amplicons

The bacterial 16S rRNA genes were amplified using extracted total DNAs in the year 2016 as matrixes with the primers Bac341F (Muyzer et al., 1993) and Bac805R (Herlemann et al., 2011) at a final concentration of 300 nM each (Table 1). For each reaction, KAPA HiFi HotStart DNA polymerase (Kapa Biosystems, Inc., Wilmington, MA, United States) at a concentration of 0.5 U was used. The matrix DNAs at a concentration of 10 ng and in a total amount of 25 µL of reaction volume were used. PCR conditions comprised an initial denaturation at 95°C for 3 min, followed by 24 cycles of denaturation (98°C for 20 s), annealing (58°C for 15 s), elongation (72°C for 30 s), and a final extension step of 72 °C for 1 min. Each reaction was performed in six repetitions that were then pooled and purified using the PCR/DNA Clean-Up Purification Kit (EURx, Gdansk, Poland). An amplicon library was sequenced on an Illumina MiSeq instrument in the DNA Sequencing and Oligonucleotide Synthesis Labolatory (oligo.pl) IBB PAS using the v3 chemistry kit in a paired-end mode.

Initial raw data quality control along with adapter trimming was performed using the fastp tool (version 0.21.0) (Chen et al., 2018). Trimmed sequences were imported into the Qiime2 software package (release 2020.11) for subsequent analysis (Bolyen et al., 2019). Read sequences were truncated (271/209 forward/reverse). The quality filtering, denoising, paired-end read merging and *de novo* chimera removal was performed using a DADA2 plugin (Callahan et al., 2016) in order to obtain Amplicon Sequence Variants (ASVs) using specified parameters (--p-min-fold-parent-over-abundance 4.00; --p-max-ee-f 2.80; --p-max-ee-r 2.80). Bacterial taxonomy was assigned for each of the ASVs using a pre-trained Naive Bayes classifier, based on the Silva 138.1 SSU database (Quast et al., 2013), which was trimmed to include only the V3–V4 region of the 16S rRNA gene, bound by Bakt_341F/Bakt_805R primer pairs. Raw sequencing data are available at the European Nucleotide Archive (ENA) under BioProject accession number PRJNA986332 and sample accession number SAMN35826646 to SAMN35826648.

### 2.5 Antibiotic resistance genes amplicons generation and sequencing

Fragments of three ARGs (namely,: *ermF*, *sul2* and *tetX*, which were identified in all stages of the purification process) were amplified from the extracted total DNAs in the year 2019 using primers ERMF-FW and ERMF-RV (Koike et al., 2010), SUL2-FW and SUL2-RW (Arabi et al., 2015) and TETX-FW and TETX-RV (Ng et al., 2001) listed in Table 1 with an attached Illumina Nextera XT adapter sequence on 3’ ends (forward adapter: TCG​TCG​GCA​GCG​TCA​GAT​GTG​TAT​AAG​AGA​CAG and reverse adapter: GTC​TCG​TGG​GCT​CGG​AGA​TGT​GTA​TAA​GAG​ACA​G). Each reaction contained primers at a final concentration of 300 nM each, and KAPA HiFi HotStart DNA polymerase (Kapa Biosystems) at a concentration of 0.5 U. A DNA matrix at a concentration of 10 ng was used, totalling 25 µL in reaction volume. The following conditions were applied for the reactions: i) for *ermF*–95°C for 3 min (1 cycle), 98°C for 20 s, 58°C for 15 s and 72 °C for 30 s (30 cycles); ii) for *sul2*–95°C for 3 min (1 cycle), 98°C for 20 s, 68.6°C for 15 s and 72°C for 30 s (29 cycles) and for iii) *tetX*–95°C for 3 min (1 cycle), 98°C for 20 s, 68°C for 15 s and 72°C for 30 s (30 cycles). Each reaction was performed in three repetitions, which then were pooled and purified using the PCR/DNA Clean-Up Purification Kit (EURx). An amplicon library was sequenced on an Illumina MiSeq instrument at the Genomed company (Poland) using the v3 chemistry kit in a paired-end mode.

### 2.6 Bioinformatic processing of raw antibiotic resistance gene amplicon data

Raw reads were subjected to quality filtering using the QIIME 2 package (release 2021.11) (Bolyen et al., 2019) prior to analysis with the same tool. During filtering, reads shorter than 100 bp were excluded from further analysis. Read sequences were truncated (290/220 forward/reverse). The quality filtering, denoising, paired-end read merging and *de novo* chimera removal was performed using DADA2 using specified parameters (-p-min-fold-parent-over-abundance 1.00; --p-max-ee-f 2.00; --p-max-ee-r 2.00). In the next step, obtained sequences and the frequency of sequences were exported from qimme artifacts to a csv table and subjected to further bioinformatic analysis. To remove artificial reads from the amplicon datasets, a BLASTN was performed against the reference sequence from the CARD database (*ermF* - ARO: 3000498, *sul2* - ARO: 3000412 and *tetX* - ARO: 3000205). Amplicon sequences for which the query coverage was below 100.00% was rejected from analysis. Raw sequencing data is available at the European Nucleotide Archive (ENA) under BioProject accession number PRJNA986332 and sample accession number SAMN35826649 to SAMN35826657.

### 2.7 Building the reference datasets of antibiotic resistance gene variants

We built the reference datasets by extracting nucleotide sequences from the NCBI NT database using specific thresholds for sequence identity and query coverage as they relate to the reference sequences. These thresholds varied. For gene *sul2* we applied 98.00% sequence identity and 98.00% query coverage. In case of gene *ermF* we used 86.00% identity and 98.00% query coverage as this was the lowest identity identified in amplicon sequences for that gene. In the case of *tetX* we applied 96.00% percent identity and 98.00% query coverage. That threshold was predicted by additional analysis which aimed to analyse the clusterisation of *tetX* variants present in the CARD database (Supplementary Table S3). The primers used were able to only amplify the *tetX* gene (ARO: 3000205). For the extension of datasets we used three reference sequences, for *ermF*–ARO:3000498, for *sul2*–ARO: 3000412 and for *tetX*–ARO: 3000205. To each sequence, additional information about taxonomic assignment was extracted by adding a -sscinames flag in the BLASTN output format. Extracted sequences in .fas format are available at the following address: http://ddlemb.com/bioinformatic-tools-and-databases/.

### 2.8 Reference sequences diversity analysis–conservation genetics

To analyse the diversity of the reference sequence of ARGs, we applied the ClustalW (Chenna et al., 2003) algorithm for the multi-sequence alignment using the default option (gap open penalty: 15.00 and gap extension penalty: 6.66) in MEGA11 software (Tamura et al., 2021). In the next step, aligned sequences were applied to create the logo consensus frequency plot using an online tool (weblogo.berkeley.edu). Aligned sequences were also used to build phylogenetic trees using the Maximum Likelihood method and Tamura-Nei model (Tamura and Nei, 1993). Initial tree(s) for the heuristic search were obtained automatically by applying the Neighbor-Join and BioNJ algorithms to a matrix of pairwise distances estimated using the Tamura-Nei model, and then selecting the topology with a superior log likelihood value. Trees were developed using MEGA11 (Tamura et al., 2021).

### 2.9 *In silico* primer pairs validation

Primer pairs used for the PCR amplicon generation were subjected to *in silico* validation using the UniPriVal software (Gorecki et al., 2019). Initial validation was aimed to assign previously described parameters, i.e., specificity (showing if the primer pair is specific to the target gene), efficacy (showing if the primer pair cover all gene variants), taxonomic efficacy (showing if the primer pair cover all putative bacterial hosts). Parameters are expressed in values from 0.00 to 1.00.

### 2.10 Calculating the diversification power of primer pairs

Based on data obtained from *in silico* validation, we applied a new calculation model which aims to estimate the diversification power of the primer pairs (DP). The DP value indicates the proportion of amplicons obtained by the particular primer pair which can be assigned to a unique reference sequence representing a given variant of the analysed gene. The DP of primer pair *i* for the amplification of ARG *j* is calculated as the ratio of the number of correct *in silico* PCR products obtained for the particular ARG using NT database as a matrix with the perfect match to only one reference sequence (*CPRSPM*_*i,j*_ *NT*) and the number of all reference sequences within the reference datasets (*RS*) of the particular ARG *j*. (1)

$${DP}_{i,j}=\frac{{CPRSPM}_{i,j}NT}{{RS}_{j}}$$ (1)

The DP parameter is expressed in values from 0.00 (none of the correct *in silico* PCR products matched the given reference sequence) to 1.00 (all of the correct *in silico* PCR products matched the specific gene variants). It should be mentioned that the formula is not working when *RS*_*j*_ value equals 1 (as in that case no variability of the reference sequences is observed).

## 3 Results and discussion

### 3.1 Occurrence of antibiotic resistance genes in wastewater samples from Oswiecim (Poland)

A qualitative PCR screening of 14 ARGs conferring resistance to aminoglycosides (one gene), MLS group (two genes), quinolones (one gene), sulfonamides (two genes) and tetracyclines (eight genes) at subsequent stages of the purification process at the WWTP in Oswiecim (Poland) was performed. The results showed that all analysed genes were found in at least one of analysed samples. The highest number of different genes (13, namely: *ermB*, *ermF*, *qrnB*, *sul1*, *sul2*, *tetA*, *tetB*, *tetC*, *tetG*, *tetM*, *tetO*, *tetT*, *tetX*) were found in primary sludge (PS). In the activated sludge (AS) seven genes (*ANT(2″)-Ia/aadB*, *ermF*, *sul1*, *sul2*, *tetC*, *tetM*, *tetX*) and in the anaerobic digestion sludge (ADS) eight (*ANT(2″)-Ia/aadB*, *ermB*, *ermF*, *sul2*, *tetC*, *tetM*, *tetT*, *tetX*) were identified. It was observed that the *qnrB*, *tetA*, *tetB* and *tetG* genes were exclusively identified in PS, while the *ermF*, *tetC*, *tetM*, *tetX* and *sul2* genes were prevalent in all samples from the WWTP (Supplementary Table S2).

The results clearly show a high prevalence of ARGs in the analysed WWTP, which stays in good agreement with previous reports (Laht et al., 2014; Karkman et al., 2016). Genes conferring resistance to aminoglycosides, macrolides, quinolones, sulfonamides and tetracyclines (including glycylcyclines) were present in at least one stage of the waste purification process. Interestingly, a retrospective analysis revealed that in 2017 (around the time of sampling) amongst the antibiotics most frequently prescribed in Poland were azithromycin (macrolide), ciprofloxacin (quinolone), sulfamethoxazole (sulfonamide) and doxycycline (glycylcycline). There was no information about the usage of aminoglycosides during antibiotic treatment (Olczak-Pieńkowska and Hryniewicz, 2021).

It is also worth mentioning that a high prevalence of the *tetX* gene was observed (identified along all stages of the tested WWTP). That gene, which confers resistance to tigecycline, is classified by the WHO as a critically important antimicrobial. Tigecycline is used for patients with serious infections in healthcare settings, who are affected by diseases for which there are very limited antimicrobial choices (WHO, 2019). Interestingly, doxycycline, which was first-line therapy around 2017, might be also modified by flavin-dependent monooxygenase encoded by *tetX* genes (Yang et al., 2004).

Results showed that the *tetX* gene and some four other genes (*ermF*, *tetC*, *sul2*, *tetM*) were prevalent along all stages of the tested WWTP. For further analyses, three of them, i.e., *ermF*, *sul2* and *tetX,* conferring resistances to various antibiotic groups, were selected.

### 3.2 Variability of ARG reference sequences

The main aim of this study was to analyse the diversity of three selected ARGs (*ermF*, *sul2* and *tetX*) applying a novel approach utilising the metagenomic analysis of PCR gene amplicons. To analyse generated ARG amplicon data, the databases of the reference sequences which gathered all variants of the analysed genes were prepared. This included 45 different variants of *ermF* gene, five variants of *sul2* gene and 37 variants of *tetX* gene, which were extracted from public repositories (Figure 2). All variants were assigned to the bacterial hosts in which they were identified. Variants of the *ermF* gene were found in 19 bacterial genera, *sul2* in 28 and *tetX* in 13. It was shown that *Acinetobacter* spp., *Bacteroides* spp., *Eschericha* spp., *Klebsiella* spp.*, Rimerella* spp. and *Salmonella* spp. are the most common genera in which the analysed ARG variants were identified. It was observed that in the case of *ermF* and *tetX* genes, with a high number of variants, 37.50% and 53.80% respectively, were identified in uncultured bacteria (Supplementary Figure S1).

The nucleotide sequence diversity of the analysed reference ARG variants differed significantly. The highest identity level was observed for the *sul2* gene, with 99.14% positions conserved. Conserved sequences cover 78.18% of the *ermF* gene variants and 87.94% of the *tetX* gene variants. It was observed that the *ermF* and *tetX* genes exhibited nucleotide polymorphism in 175 and 141 different loci, respectively. Differences are distributed unequally along the whole sequence (Supplementary Figures S3, S5). The *sul2* gene exhibited nucleotide polymorphism in only 7 loci, which are located at the beginning and the end of the sequence (Supplementary Figure S4).

### 3.3 Statistics of primer pairs used for generation of ARG amplicon data

**FIGURE 1:**
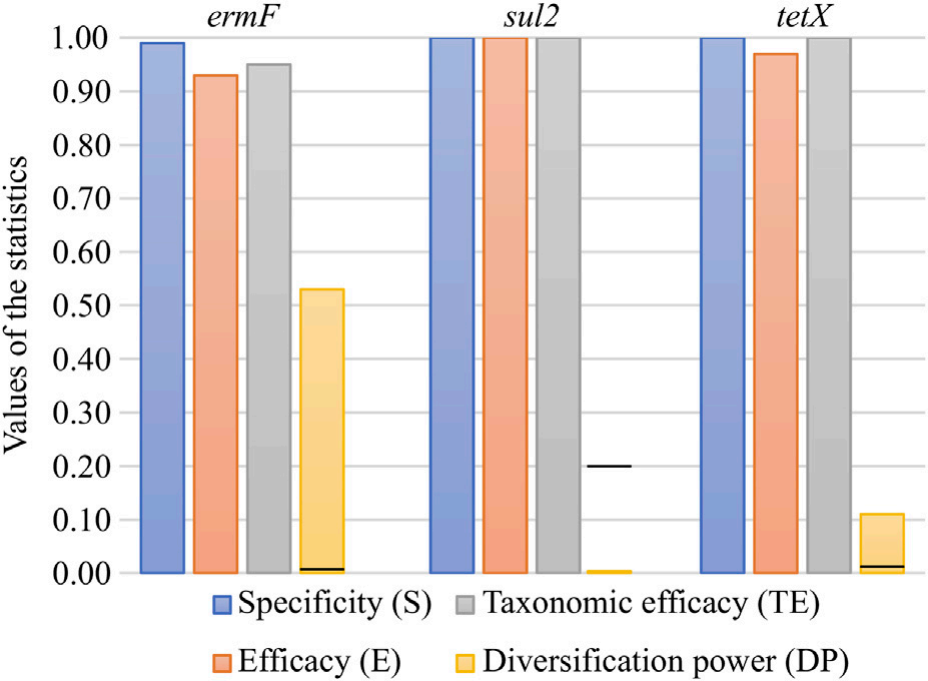
*In silico* validation of the specificity, efficacy, taxonomic efficacy and diversification power of the primer pairs used for ARG amplicon generation. The black lines on the DP bars correspond to the first value greater than 0 for the given set.

*In silico* validation revealed that all three primer pairs used for PCR amplicon generation were specific to the analysed ARGs. The primer pairs exhibited very high parameters, ranging from 0.93 to 1.00 for efficacy (E) and from 0.95 to 1.00 for taxonomic efficacy (TE). These results indicated that the primer pairs used were sufficient in the general screening of ARGs in the environmental samples (Figure 1). The primer pairs varied significantly when calculating the novel parameter, developed in this study, i.e., the diversification power (DP) value. It was shown that the primer pair for the *ermF* gene was distinguishing only slightly over half of all variants (DP = 0.53). It was the highest DP value obtained for the analysed set of primer pairs. The DP value equal to 0.11 was observed for the primer pair for the *tetX* gene. Analysis showed that none of variants can be distinguished by primer pairs used for the *sul2* gene, since the primer pair amplifies a highly conserved (identical in all variants) DNA fragment (DP = 0.00) (Figure 1). The analysis performed indicated that high divergence between DP values was the result of the region which was amplified by the primer pairs used. It was shown that in the case of the *sul2* and *tetX* genes, the majority of single nucleotide polymorphisms (SNPs, distinguishing between variants) were present outside of the amplified region (Supplementary Figures S4, S5). To maximise the DP value of the primer pair, it should be designed within the region showing high variability but shared between the highest possible number of variants.

It should be remembered that increasing the value of DP has limitations. The currently used preparation kits for the construction of the Illumina library make it possible to generate pair-end amplicon reads of maximal length, reaching 600 base pairs (bp). Therefore, in the case of large genes, it might be unfeasible to cover the whole variability by using only one primer pair.

### 3.4 Antibiotic resistance gene variants drift along subsequent stages of the WWTP

**FIGURE 2:**
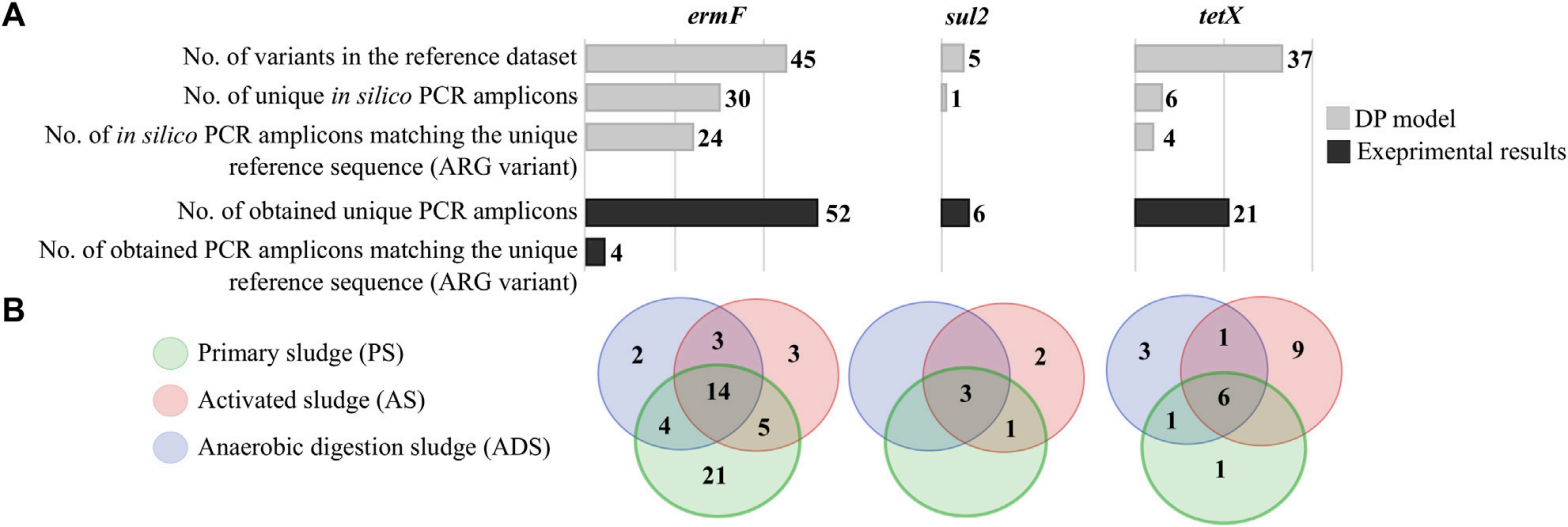
Occurrence and distribution of the *ermF*, *sul2* and *tetX* gene variants in the Oswiecim WWTP. **(A)**, Number of different variants calculated by the diversification power (DP) model and obtained from the laboratory experiments. (**B)**, Distribution of the unique variants obtained from the laboratory experiments along subsequent stages of the purification process in the WWTP.

The model used for calculations indicated the actual number of various amplicons that might be theoretically obtained by a particular primer pair. According to calculations, a maximum of 30 unique PCR amplicons, characteristic for 24 reference sequences (gene variants) could be obtained for the *ermF* gene. Six out of 30 unique PCR amplicons were identical with two or more reference sequences. In total, 45 variants of the *ermF* gene were distinguished so far, as revealed by an analysis of public databases. In the case of the *sul2* gene, only one unique PCR amplicon should be observed. This amplicon cannot be assigned to a particular variant as the region amplified by the primer pairs is identical in all (six) variants. For the *tetX* gene, in total 37 variants were identified in the NT database. According to the DP model, the PCR primer pair used for *tetX* amplicon generation should amplify a maximum of six unique amplicons, from which only four could be assigned to a unique reference sequence (Figure 2A).

The primer pairs described have been utilized for the PCR amplicon generation using total DNA matrices isolated from three WWTP samples from subsequent stages of the waste purification process. The total of 385,809 paired-end reads was obtained from the DNA sequencing run. Average reads count per sample was 128,603 reads (min. 109,845, max. 149,021). In the course of the quality control, which removed low quality and unspecific reads, the average number of reads per sample dropped to 140,870 (min. 49,229, max. 183,069). It resulted in obtaining 52 unique *ermF* amplicons, six amplicons of the *sul2* gene and 21 different amplicons of the *tetX* gene. An analysis of the results obtained showed that only four out of 79 generated amplicons can be assigned to a unique reference sequence. Other PCR amplicons obtained showed identity with more than one variant collected in the reference dataset. In all cases, the number of unique amplicons obtained is greater than the number of unique PCR products predicted by *in silico* calculations (Figure 2A). Assuming the sequencing was performed correctly to the highest degree possible, it may be stated that the novel variants of the analysed ARGs have been identified. However, here one has to be critical as it was described that next-generation sequencing (NGS) may generate errors. The substitution error rate by conventional NGS techniques is reported to be around >0.10% (Goodwin et al., 2016; Salk et al., 2018; Ma et al., 2019). In the case of the Illumina MiSeq platform which has been applied in this study, the median error rate is 0.47% (Stoler and Nekrutenko, 2021).

Around 28.00% ± 0.01% of identified variants were identified in samples originating from all stages of the analysed WWTP. It was observed that the highest variability of the *ermF* variants (84.60% of all identified variants) occurred in the primary sludge sample (PS), whereas in the case of the *sul2* and *tetX* amplicons, the highest variability was observed in the activated sludge samples (AS). The results showed that anaerobic digestion sludge (ADS) reduces the number of unique variants of the *ermF* and *sul2* genes (Figure 2B).

### 3.5 Matching antibiotic resistance gene amplicons to reference variants

**FIGURE 3:**
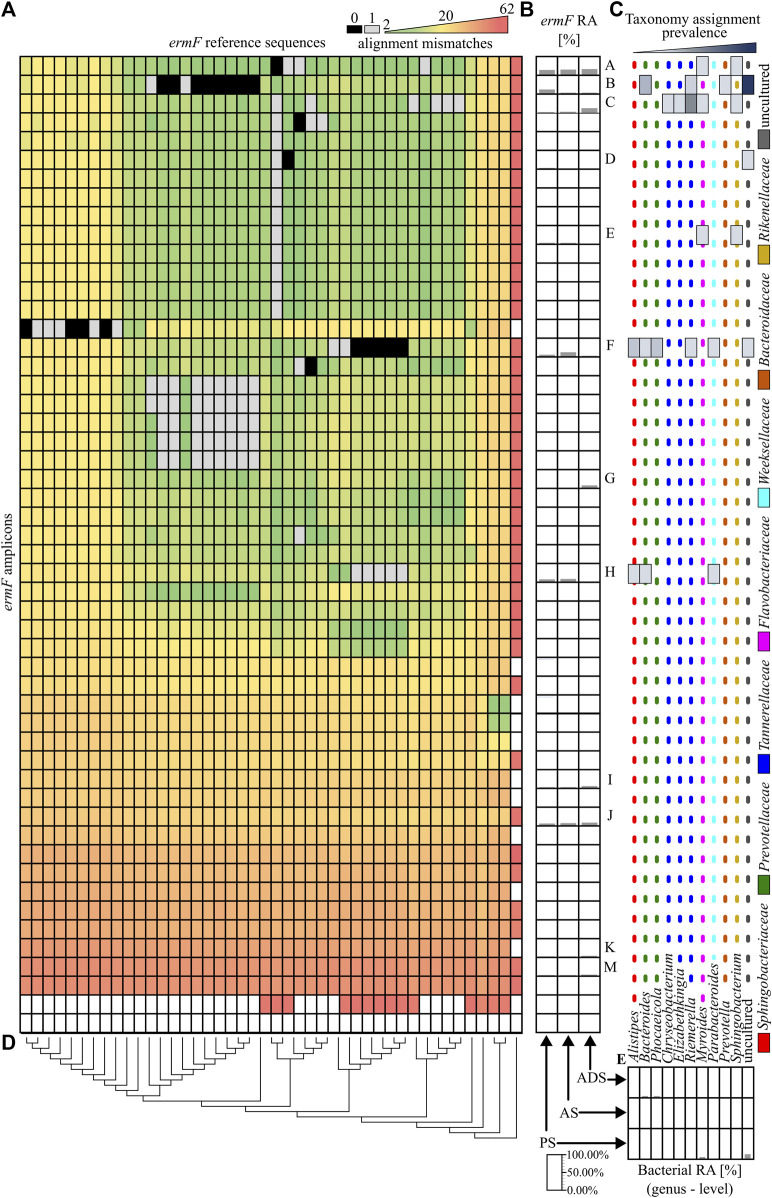
Analysis of the *ermF* amplicons. **(A)**, A heat map showing the similarity of obtained PCR amplicons to the reference sequences, based on the mismatch value from the BLASTN output. The black colour indicates 0 mismatches and the grey colour, 1 mismatch. **(B)**, The relative abundance of the *ermF* amplicons in the different stages of the purification process. The *ermF* amplicons with an abundance higher than 1.00% in any of the samples have been marked with a unique capital letter (from A to M). **(C)**, The hosts of the *ermF* gene for which the given amplicon has a perfect match (0 mismatches or 1 mismatch) according to NCBI NT database. The dotted, coloured lines indicate different families of the hosts. **(D)**, The phylogenetic tree, representing the homology of the reference sequences. **(E)**, The relative abundance of the bacterial community in the analysed samples. Abbreviations: RA–relative abundance, PS–primary sludge, AS–activated sludge, ADS–anaerobic digestion sludge.

**FIGURE 4:**
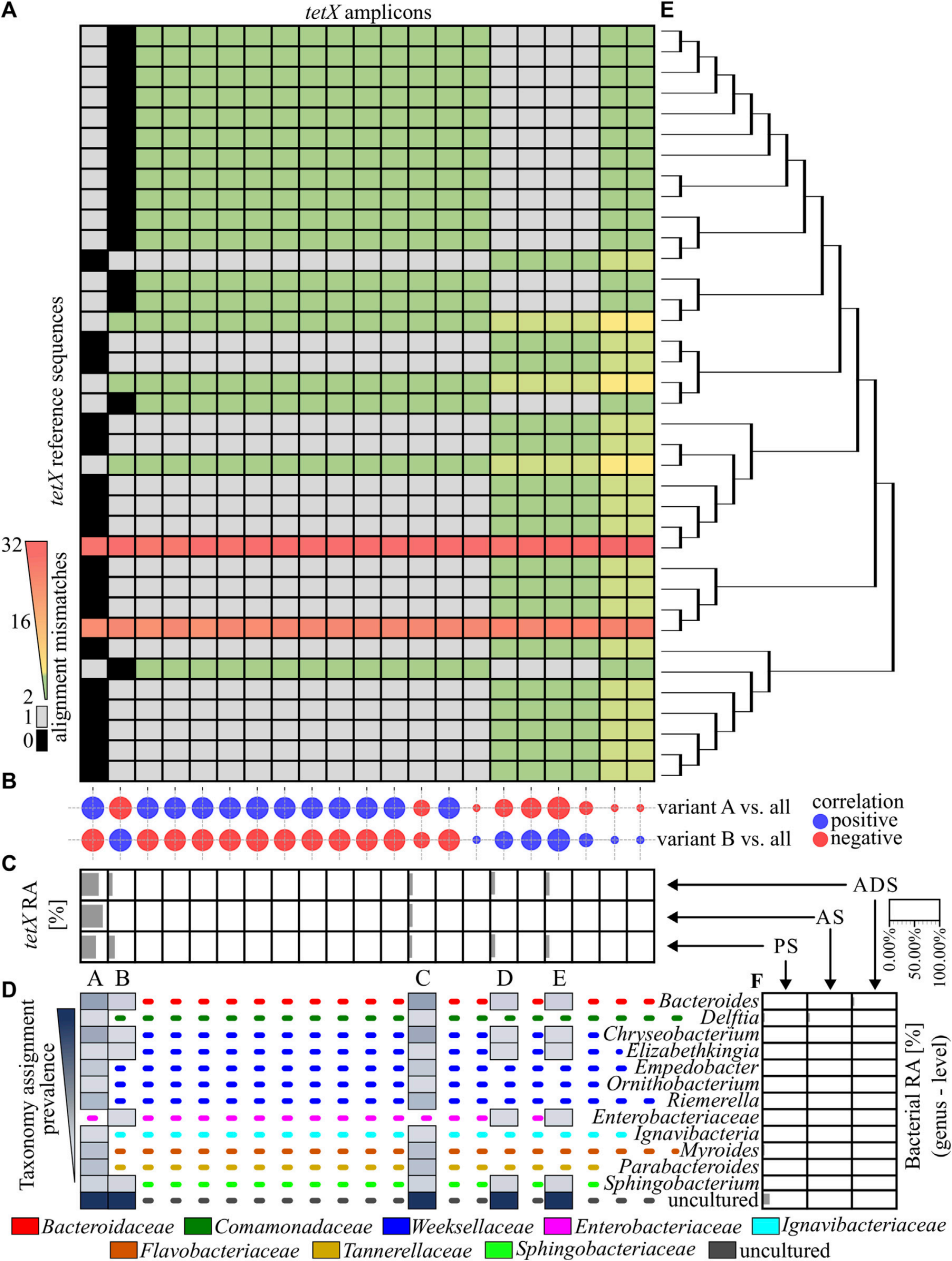
Analysis of the *tetX* amplicons. **(A)**, A heat map showing the similarity of the PCR amplicons obtained to the reference sequences, based on the mismatch value from the BLASTN output. The black colour indicate 0 mismatches and the grey colour, 1 mismatch. **(B)**, The correlation plot, showing the similarity of the relative abundance pattern for a particular *tetX* amplicon between the analysed samples. The size of the bubbles correspond to *R* ^2^ values; the red colour is a negative correlation and the blue colour is a positive correlation. **(C)**, The relative abundance of the *ermF* amplicons in the different stages of the purification process. The *tetX* amplicon with an abundance higher than 1.00% in any of the samples have been marked with a unique capital letter (from A to E). **(D)**, The host of the *tetX* gene for which the given amplicon has a perfect match (0 mismatches or 1 mismatch) according to NCBI NT database. The dotted, coloured lines indicate different families of the listed hosts. **(E)**, The phylogenetic tree representing the homology of the reference sequences. **(F)**, The relative abundance of the bacterial community in the analysed samples. Abbreviations: RA–relative abundance, PS–primary sludge, AS–activated sludge, ADS–anaerobic digestion sludge.

In the next step, the similarity of obtained ARG amplicons to reference sequences of variants was analysed. It was revealed that 11 out of 79 identified amplicons perfectly matched one or more reference sequences. From those, only four amplicons of the *ermF* gene could be assigned to the unique reference sequence (variant). It was observed that the vast majority of *ermF* amplicons and two of the *tetX* amplicons had at least two mismatches with known reference sequences. The maximum dissimilarity between the amplicons obtained and the reference sequences reached up to 50 mismatches (Figure 3A and Figure 4A).

The relative abundance of obtained amplicons in subsequent stages of the WWTP has been analysed. It was shown that the relative abundance of 12 *ermF* amplicons, five *tetX* amplicons and one *sul2* amplicon was higher than 1.00% in at least one of the analysed samples, and these dominant variants constituted on average 97.97% ± 1.61% of all obtained amplicons in the analysed sample (Figure 3B and Figure 4C).

Within the highly abundant PCR amplicons of the *ermF* gene, only two (A and D) can be assigned to a unique reference sequence. Variant A maintains high abundance along subsequent stages of the analysed WWTP (35.49% ± 3.00%). It was shown that these amplicons are identical to the homologous region of the reference sequences which previously were identified in *Myroides* spp., *Sphingobacterium* spp. and numerous undefined non-cultivable bacteria. The highest relative abundance was observed for amplicon F of the *ermF* gene. The amplicon reached 44.22% in the AS sample. It was shown that the amplicon is identical to the homologous region of five different reference sequences which were previously identified in *Alistipes* spp., *Bacteroides* spp. *Parabacteroides* spp., *Phocaeicola* spp. and *Rimieralla* spp. and some uncultured bacteria. However, it is important to mention that the high relative abundance of some amplicons might be the result of a multiplication signal from different variants of a given ARG that could not be distinguished by the primer pairs used. The largest shift of the abundance of a specific *ermF* amplicon was observed for amplicon C, for which a significant change between aerobic samples (PS and AS) and anaerobic sample (ADS) was observed. Here, the relative abundance increased from 5.30% ± 1.33% (PS and AS) to 29.52% (ADS). Amplicon C did not show a perfect match to any of the reference sequences. Eventually, it was shown that there are some completely novel variants present in the analysed WWTP. The relative abundance of the amplicons marked as I, J, K, M fluctuate from 0.42% to 11.84%. Those amplicons exhibited at least 13 mismatches to the closest reference variant of the *ermF* gene. This indicates that there is a pool of unexplored variants of ARGs present in the WWTP (Figures 3A–C).

Amplicons of the *tetX* gene were equally similar to many reference sequences. It was shown that only two out of the 21 amplicons obtained perfectly matched to the reference sequences. The amplicon marked A matched to 16 reference sequences which were identified in 12 genera belonging to eight bacterial families. Amplicon B matched to 14 another reference sequences. These *tetX* gene variants were previously identified in six different genera belonging to five bacterial families. The relative abundance of amplicon A was dominating and reached 63.45% in PS, 93.91% in AS and 75.65% in ADS (Figures 4A, B, D).

In the case of the *sul2*, one out of six obtained amplicons matched to all reference sequences. Other PCR amplicons exhibited a single mismatch to the reference sequences. Additionally, it was observed that the amplicon with the perfect match significantly dominated in the analysed WWTP. The relative abundance of this amplicon reached 99.52% ± 0.26% (Supplementary Figures S2A, B). It is worth mentioning that the DP value of the primer pair used for amplification of the *sul2* gene was 0.00, which means that only one sequence should be observed. It is highly probable that other observed amplicons of the *sul2* gene were generated via sequencing errors or these are truly novel variants. However, the contribution of these amplicons in the samples are scarce (less than 0.20%), and the dissimilarity to reference sequences (1 mismatch) is located in random loci. A similar conclusion might be derived from the results obtained for the *tetX* analysis. That primer pair also exhibited a very low value of DP (0.11), showing that only four unique sequences should be observed (Figure 2).

Overall result indicates that the tested WWTP (and other WWTPs by default) are reservoirs of many different alleles of the genes encoding the same mechanism of resistance to antibiotics. It was shown that some of identified variants may persist along the whole purification process at the significant abundance. It was observed for variants A, C, K, M of the *ermF* gene or variants A and C of the *tetX* gene. Results showed that some of these variants are present in more than one host assigned to different bacterial families (Figures 3B–D). It suggest that those particular variants might be present on mobile genetic elements and can be easily spread between different type of bacteria (Che et al., 2019). We also observed that some of the identified variants change their abundance along the process, e.g., variant B or C of the *ermF* gene. It indicated that some of gene variants might be highly strain specific (Van Meervenne et al., 2012; Rosconi et al., 2022). The changes of the conditions at subsequent stages of the purification process impacted the community structure which might results in the reduction or enrichment of the particular ARGs variants. It might be a possible explanation for the enrichment of the variant C in ADS stage where level of oxygen is relevantly reduced (Guo et al., 2015). Lastly, we observed the situation where variant was present at high abundance on the first stage of the process and then declined (variant B of the *ermF* gene).

### 3.6 Prediction bacterial hosts of the generated antibiotic resistance gene amplicons

To predict the potential host of identified ARG amplicon variants, a taxonomic analysis of the WWTP samples was performed. The total of 2,069,058 paired-end reads was obtained from the DNA sequencing run. Average reads count per sample was 229,895 reads (min. 162,918, max. 302,738). In the course of the quality control, which removed low quality and unspecific reads, the average number of reads per sample dropped to 22,899 (min. 17,985, max. 29,466).

Previously, it was shown that some of the obtained amplicons might be assigned to a specific gene variant, perfectly matching one or more reference sequences. However, the taxonomic analysis performed showed that the majority of genera in which these variants were previously identified were not present in the Oswiecim WWTP. Only five out of 42 genera previously assigned to the analysed ARGs were identified in the WWTP samples that were analysed. Those include representatives of *Acinetobacter* spp., *Aeromonas* spp., *Bacteroidestes* spp., *Ignavibacteria* spp., and *Prevotella* spp. However, even though the above-mentioned ARG amplicons are prevalent in the WWTP samples, the relative abundance of the listed strains were very low (form 0.05%–1.12%), and these genera were detected in a maximum of two out of three stages of the purification process (Figure 3E and Figure 4F). This suggested that some of the ARG variants already described in reference databases might be present in novel hosts to which they were currently unassigned.

Finally, an attempt was made to assign the hosts by analysing the correlation of relative abundance changes along the process between obtained ARGs amplicons and the present taxa. It is worth mentioning that the analysis could produce reliable results only in the case of amplicons to which only one reference sequence was assigned. In other cases, the relative abundance might be factitiously enhanced by multiplication of abundance by different variants which were not distinguished by the primer pair used. Therefore, for this analysis, only primer pairs with the highest possibly DP should be utilised. Only four amplicons generated for the *ermF* gene from which two exhibited substantial relative abundance fulfilled the rule. It was shown that amplicon A exhibited strong correlation (*R* ^2^ = 0.93 ± 0.07) with six undefined genera, and amplicon D exhibited a positive correlation with an unspecified taxon belonging to the *Anaerolineae* class.

## 4 Conclusion

• The analysis of ARGs amplicons is sufficient method for ARG variant diversity and dynamics analyses. However, it needs the usage of primer pairs with possibly the highest diversification power value, which enhance the chance to identify all present variants and assign identified amplicons to potential hosts. In this study we proposed an approach which shown to be effective in selection of the most suitable primer pairs for such analyses.
• Further development of the method should focus on: i) the removal of the subclonal variants generated by sequencing errors, to be sure that identified variants are not the sequencing artifacts; ii) further extension of the reference datasets with special attention to the assignment of bacterial hosts. A better taxonomic assignment of ARG variants may be a crucial point for better removal of AMR via host eradication by setting harmful conditions for the specific groups of bacteria.
• The diversity of the genes encoding the same mechanism of resistance to antibiotics in much more complex than expected. There are still a large pool of ARG variants which are not identified and described in details.
• The wastewater treatment processes have a strong impact on the diversity of the ARG variants. The basis of that phenomenon is not well understood and needs further considerations.

## Data Availability

The datasets presented in this study can be found in online repositories. The names of the repository/repositories and accession number(s) can be found in the article/Supplementary Material.
