# Supplementary material for: Diversity of antibiotic resistance gene variants at subsequent stages of the wastewater treatment process revealed by a metagenomic analysis of PCR amplicons
Source: Front Genet. 2024 Jan 11;14:1334646. doi: 10.3389/fgene.2023.1334646 (PMC10808613; doi:10.3389/fgene.2023.1334646)
Supplement: Supplementary file 1 [file Presentation1.pdf]

Supplementary material

Table S1. Table showing impact of N-value of RSj on first value greater than 0 in the set.

N-value of RSj	2	3	4	5	6	7	8	9	10	15	20	50	100	1000
First value >0 in the set	0.50	0.33	0.25	0.20	0.17	0.14	0.13	0.11	0.10	0.07	0.05	0.02	0.01	0.001

Table S2. Antibiotic resistance genes – screening results

	aadB	*ermB*	*ermF*	*qrnB*	*sul1*	*sul2*	*tetA*	*tetB*	*tetC*	*tetG*	*tetM*	*tetO*	*tetT*	*tetX*
Primary sludge		+	+	+	+	+	+	+	+	+	+	+	+	+
Activated sludge	+		+		+	+			+		+			+
Anaerobic digestion sludge	+	+	+			+			+		+		+	+

Table S3. Table showing identity level between different variants of the *tet(X)* gene in the CARD database

	Tet(X1)	*Tet(X3)*	*Tet(X4)*	*Tet(X5)*	*Tet(X6)*	*tetX*
Tet(X1)	100.00					
Tet(X3)		100.00	87.07	86.29	82.23	87.35
Tet(X4)		87.07	100.00	95.50	91.86	95.14
Tet(X5)		86.29	95.50	100.00	96.31	91.73
Tet(X6)		82.23	91.86	96.31	100.00	87.75
tetX		87.35	95.14	91.73	87.75	100.00

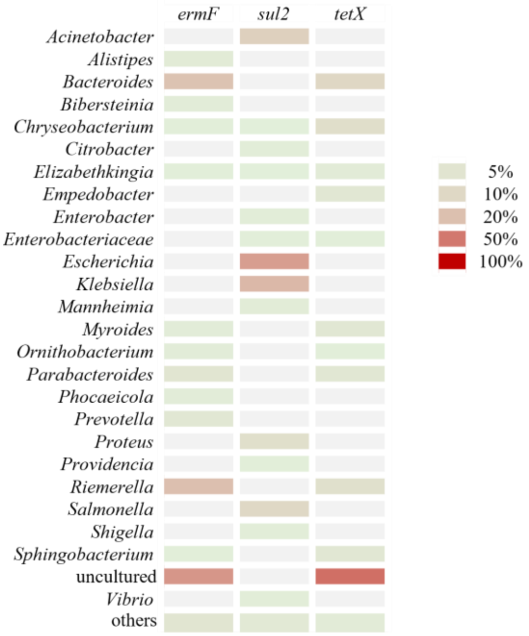

Figure S1. Taxon distribution between analysed antibiotic resistance genes

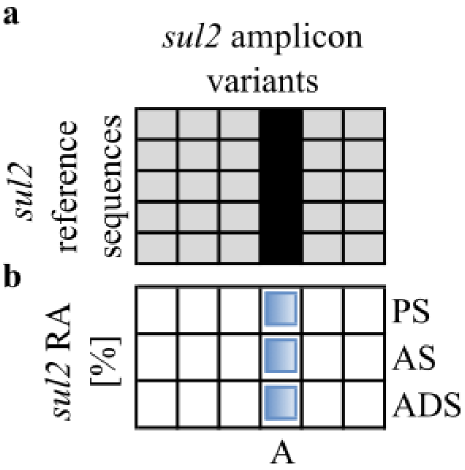

Figure S2. Analysis of the *sul2* amplicons. **a**, A heat map showing the similarity of obtained PCR amplicons to the reference dataset based on mismatch value from the BLASTN output. The black colour indicates 0 mismatches and the grey colour, 1 mismatch. **b**, The relative abundance of the *ermF* amplicons along different stages of the purification process. Variants with an abundance higher than 1.00% in any of the samples have been marked with a unique capital letter (A).

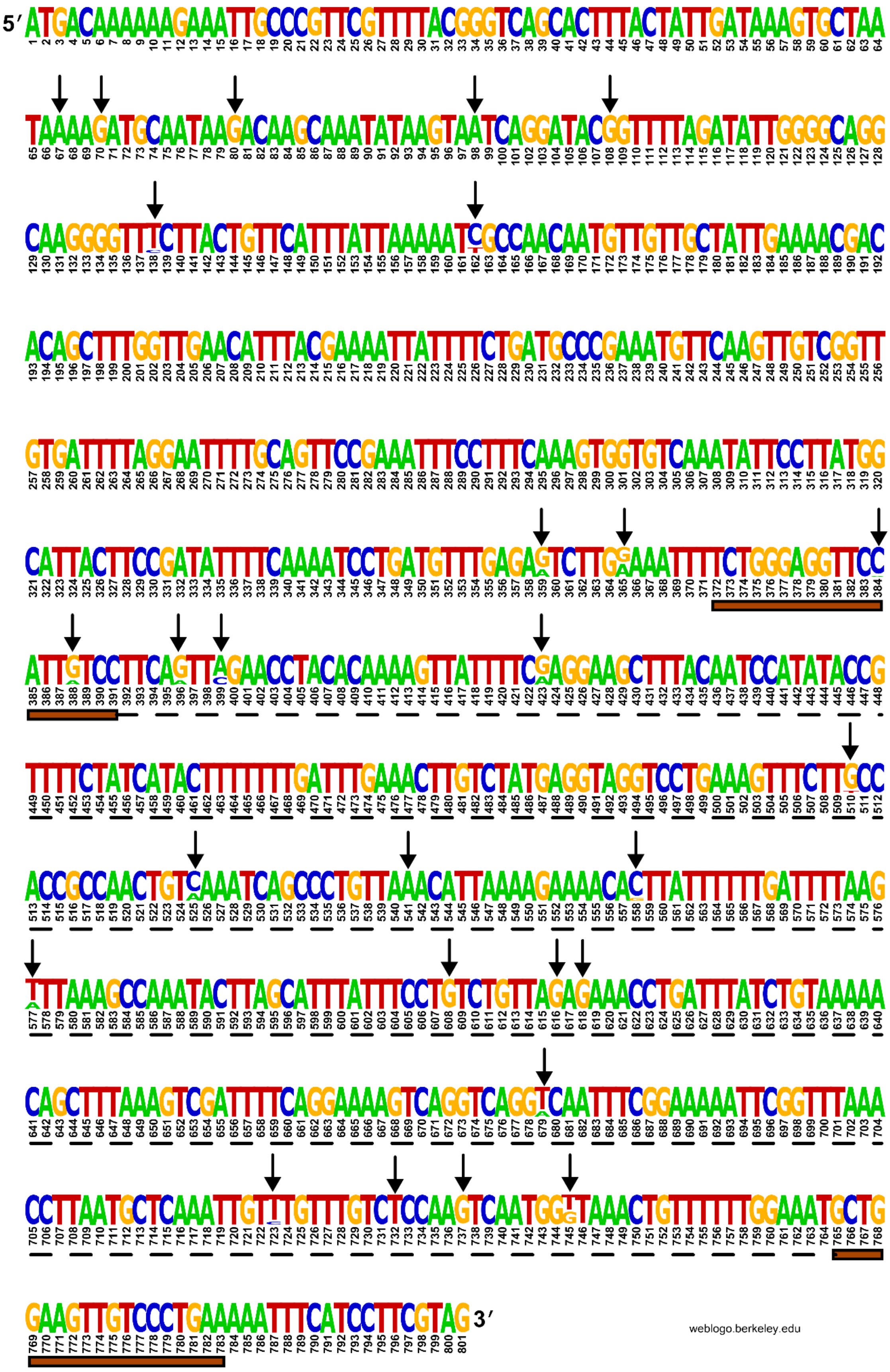

Figure S3. Logo consensus generated for the *ermF* gene variants. The brown blocks represent sites for primers. The dashed line corresponds to the amplified region. The black arrows indicate loci with variable nucleotides. The occurrence of a particular nucleotide in a particular position corresponds to the size of the letter in the position.

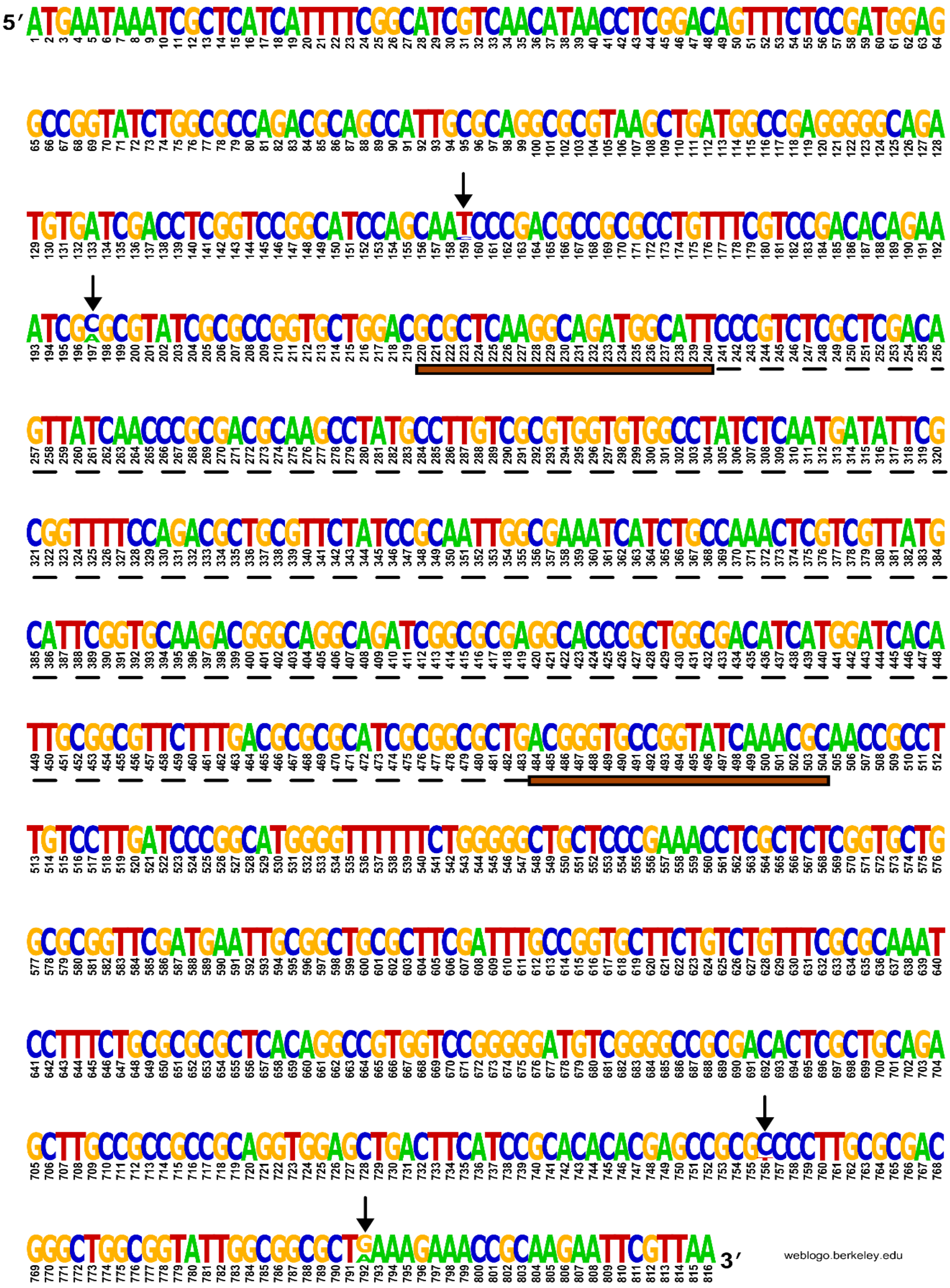

Figure S4. Logo consensus generated for the *sul2* gene variants. The brown blocks represent sites for primers. The dashed line corresponds to the amplified region. The black arrows indicate loci with variable nucleotides. The occurrence of a particular nucleotide in a particular position corresponds to the size of the letter in the position.

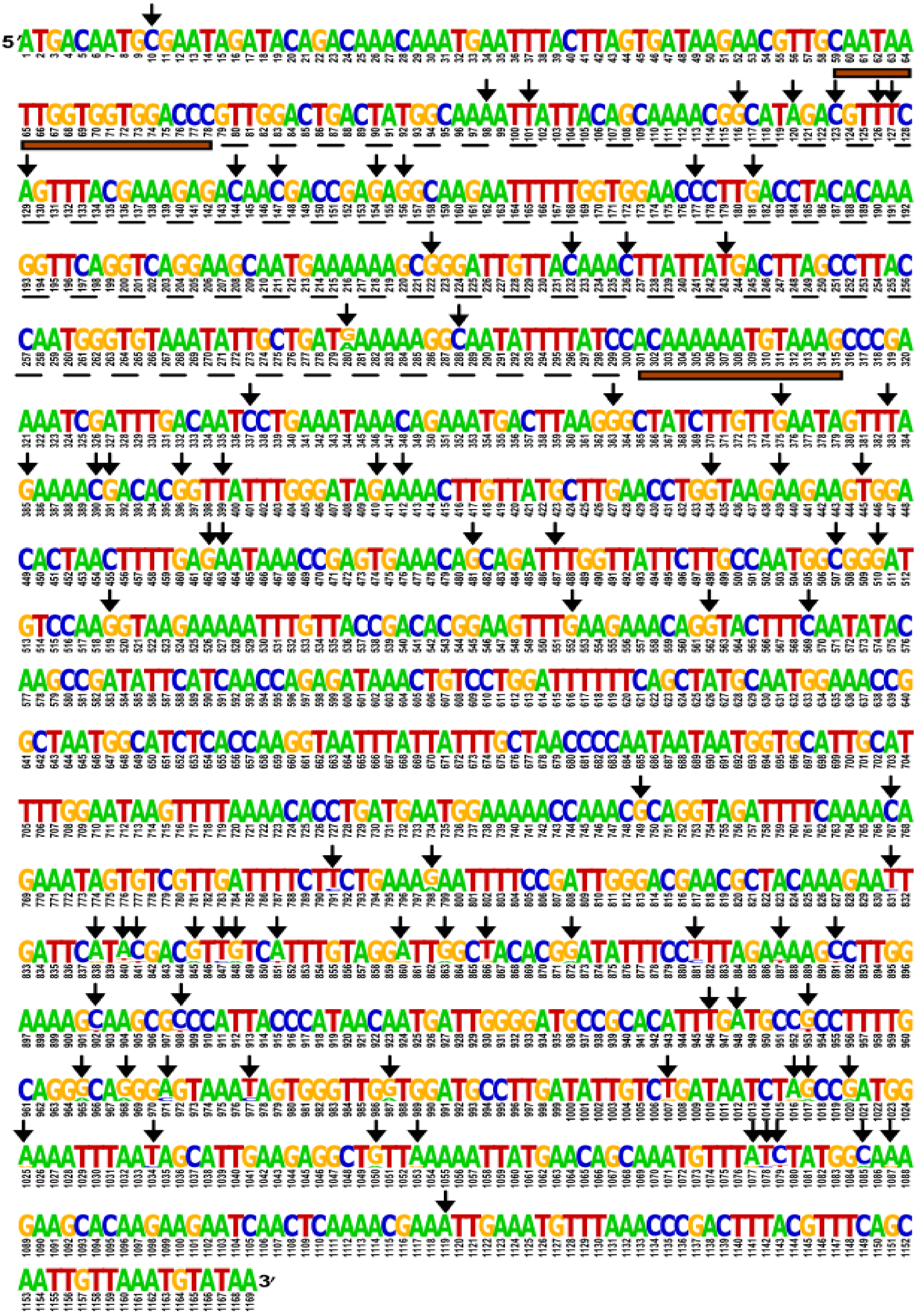

Figure S5. Logo consensus generated for the *tetX* gene variants. The brown blocks represent sites for primers.

The dashed line corresponds to the amplified region. The black arrows indicate loci with variable nucleotides. The occurrence of a particular nucleotide in a particular position corresponds to the size of the letter in the position.
